# One-year changes in body composition and phase angle during feminizing gender-affirming hormone therapy: a longitudinal and comparative study

**DOI:** 10.3389/fendo.2026.1694472

**Published:** 2026-03-12

**Authors:** Suna Avcı, Zehra Kara, Emre Durcan, Özge Polat Korkmaz, Ali Kimiaei, Seyedehtina Safaei, Şenol Turan, Alper Döventaş, Deniz Suna Erdinçler, Pınar Kadioglu

**Affiliations:** 1 Istanbul Universitesi-Cerrahpasa Cerrahpasa Tip Fakultesi, Fatih, Türkiye; 2 Hatay Mustafa Kemal University Hospital, Hatay, Türkiye

**Keywords:** assigned male at birth, body composition, gender dysphoria, gender-affirming hormone therapy, metabolic changes, phase angle

## Abstract

**Background:**

Gender dysphoria (GD) refers to the clinically significant distress or discomfort that may arise from a marked incongruence between an individual’s gender identity and the sex assigned at birth, involving primary and/or secondary sex characteristics and social roles. Gender-affirming hormone therapy (GAHT) aims to alleviate this distress by aligning secondary sex characteristics with gender identity and improving psychological well-being. This study examined the effects of GAHT on metabolic and body composition changes in assigned male at birth (AMAB) individuals.

**Methods:**

This single-center longitudinal study (2019–2022) followed 20 GAHT-naïve AMAB individuals with gender dysphoria, reassessed after 12 months of GAHT, with 24 cisgender women serving as a reference group. GAHT included estradiol with cyproterone acetate or spironolactone. Outcomes included bioelectrical impedance analysis (BIA)-derived phase angle (PhA) and body cell mass, as well as handgrip strength and gait speed.

**Results:**

After 12 months of GAHT, AMAB participants receiving hormone therapy showed significant reductions in fat-free mass (49.9 [39.4–54.6] kg, p = 0.01), fat-free mass index (FFMI) (18.4 [16.5–19.4] kg/m², p = 0.04), body cell mass (25.7 [20.6–29.3] kg, p = 0.04), and phase angle (6.9 [6.0–7.6], p = 0.04). Fat mass increased from 10.6 [8.9–13.8] to 12.9 [10.8–16.1] kg, although this change did not reach statistical significance (p = 0.07). Grip strength showed a non-significant decline (32.5 ± 6.6 kg, p = 0.20), while gait speed remained stable (1.52 [1.30–1.80] m/s, p = 0.60). Despite these changes, lean mass indices and phase angle remained higher than in cisgender women at follow-up (all p < 0.001). Hormonal and metabolic markers remained stable over the one-year follow-up.

**Conclusion:**

During the first year of GAHT, AMAB participants exhibited notable physiological changes, including decreases in fat-free mass (FFM) and phase angle. Because phase angle is an indicator of cellular integrity and muscle quality, its decline during GAHT warrants careful monitoring in future long-term studies. These findings underscore the importance of comprehensive body composition assessments in AMAB individuals receiving feminizing GAHT. Further studies with longer follow-up periods and larger cohorts are needed to elucidate the long-term clinical significance of these changes.

## Introduction

Gender refers to the sociocultural roles and expectations assigned to individuals based on their biological characteristics, whereas gender identity describes a person’s internal sense of their gender (1, 2). Gender-affirming hormone therapy (GAHT) supports alignment between physical characteristics and gender identity and has demonstrated beneficial effects on psychological well-being (3, 4). By inducing physical changes consistent with gender identity, GAHT contributes significantly to the alleviation of gender dysphoria (GD) (5).

In individuals assigned male at birth (AMAB) diagnosed with gender dysphoria, feminizing GAHT typically involves estrogen therapy combined with antiandrogens to suppress testosterone (6). This regimen promotes secondary sex characteristics, including breast development and skin softening, while facilitating a shift toward female-typical fat distribution. Concomitantly, reported reductions in muscle strength, lean body mass (LBM), and muscle cross-sectional area contribute to a more feminine body composition (7, 8).

However, GAHT may exert long-term effects on several physiological systems, including cardiovascular health, bone mineral density (BMD), and metabolic function; these areas remain active fields of investigation (9). Despite growing interest in transgender health, significant gaps remain in understanding how GAHT influences muscle composition, physical function, and long-term body composition outcomes.

During feminizing hormone therapy, monitoring changes in muscle and body composition is an essential component of comprehensive care. AMAB individuals undergoing feminizing GAHT typically experience reductions in muscle mass and strength. This is an expected physiological adaptation that reflects the alignment of physical characteristics with their gender identity (8, 10, 11). By establishing baseline measurements and tracking these shifts, healthcare providers can ensure that these adaptations occur within healthy parameters.

Tools such as bioelectrical impedance analysis (BIA), which includes phase angle (PhA) measurements, offer non-invasive ways to monitor these physiological changes (12). GAHT in AMAB individuals can significantly impact muscle mass and strength, leading to body composition changes that align with female physiological patterns. Studies have shown that estrogen-based treatments are associated with reductions in LBM and muscle strength, which may influence overall muscle health over time (10, 13, 14). Although these changes represent anticipated components of feminizing adaptation, longitudinal monitoring remains important to ensure that muscle-related parameters remain within clinically appropriate ranges over time.

Accordingly, this longitudinal study tracked AMAB individuals over 12 months of GAHT to assess within-subject physiological changes. Beyond conventional markers of muscle mass and strength, we assessed the utility of advanced bioimpedance-derived parameters, including phase angle (PhA) and body cell mass (BCM). The study aimed to characterize the timeline and magnitude of body composition shifts, thereby informing evidence-based strategies for monitoring in gender-affirming care.

## Materials and methods

### Participants and study procedure

This study combined two methodological approaches: a cross-sectional comparison with cisgender women and a 12-month longitudinal follow-up of individuals assigned male at birth (AMAB) receiving gender-affirming hormone therapy (GAHT). Accordingly, the longitudinal component should be interpreted as a within-subject analysis of AMAB participants, with cisgender women serving as a cross-sectional reference group rather than a longitudinal control. AMAB participants were assessed at GAHT initiation and reassessed after 12 months to capture within-subject changes over time. The research was conducted at the Endocrinology, Metabolism, and Diabetes Outpatient Clinic of Istanbul University-Cerrahpaşa, Cerrahpaşa Medical School, between 2019 and 2022.

Participants were categorized into three groups for analysis:

- Group 1: Cisgender women (control group, n = 24), recruited from a general internal medicine clinic. All participants were in good general health, non-smokers, and free of known endocrine, metabolic, or chronic systemic diseases. None were using hormonal contraceptives. Blood sampling was performed during the early follicular phase (cycle days 2–3) to minimize hormonal variability.
- Group 2: AMAB participants assessed at GAHT initiation (GAHT-naïve) (n = 20).
- Group 3: The same AMAB participants reassessed after 12 months of GAHT (n = 20). Thus, longitudinal analyses represent paired within-subject assessments of the same individuals.

Demographic characteristics, comorbidities, medication histories, and polypharmacy status were recorded. All AMAB participants received standardized dietary counseling and lifestyle guidance. Clinical assessments, including hormone monitoring, were scheduled at approximately three-month intervals in accordance with guideline-based GAHT follow-up protocols.

### Inclusion and exclusion criteria

The study included AMAB individuals aged 18–45 years with a diagnosis of gender dysphoria (GD) established according to the *Diagnostic and Statistical Manual of Mental Disorders, Fifth Edition* (DSM-5) within one month prior to initiating GAHT. Participants were required to be in stable general health.

Exclusion criteria included obesity (body mass index [BMI] ≥ 30 kg/m²), metabolic or endocrine disorders (including diabetes mellitus, thyroid dysfunction, or chronic kidney/liver disease), malnutrition, or any condition likely to influence body composition. Individuals using anabolic steroids or corticosteroids, and those with prior exposure to any hormonal therapy, were also excluded.

### Measurements

All assessments were performed by the same trained investigator to minimize inter-observer variability.

- Anthropometry: Height (cm), weight (kg), BMI (kg/m²), and circumferences (waist, hip, arm, and calf) were obtained using standard clinical procedures.
- Muscle Strength: Handgrip strength was evaluated using a Jamar hydraulic hand dynamometer. Participants were seated with elbows supported and arms flexed at a 90° angle. The test was performed three times, and the highest value was recorded.
- Bioelectrical Impedance Analysis (BIA): Body composition was assessed using multi-frequency BIA with the Bodystat Quadscan 4000^®^. Measurements were performed after a 12-hour fast with participants in the supine position. Electrodes were placed on the dorsal surfaces of the hands and feet. The device provided estimates of fat-free mass (FFM), fat mass, body cell mass (BCM), and related indices, including the fat-free mass index (FFMI) and body fat mass index (BFMI). Phase angle (PhA) was calculated automatically from resistance and reactance values.
- Gait Speed: A 6-meter walk test was used to evaluate physical function. Participants walked at a comfortable pace, and the time required to complete the distance was recorded via a stopwatch.

### Laboratory measurements and hormone therapy protocol

Fasting venous blood samples were collected at GAHT initiation and at the 12-month follow-up. Serum was separated via centrifugation and stored at –20 °C. To minimize inter-assay variation, all samples from each participant were analyzed within the same batch. Hormonal measurements, including estradiol, total testosterone, luteinizing hormone (LH), follicle-stimulating hormone (FSH), and prolactin, were assessed using an electrochemiluminescence immunoassay (ECLIA) on a Roche Cobas e602 analyzer.

Target hormone levels were established according to the Endocrine Society (2017) and the World Professional Association for Transgender Health (WPATH) Standards of Care Version 8 (2022) guidelines, aiming for estradiol concentrations of 100–200 pg/mL and total testosterone < 50 ng/dL. Feminizing therapy primarily consisted of oral estradiol valerate, initiated at 2 mg/day and titrated as necessary. Antiandrogen treatment included cyproterone acetate (typically 50 mg/day) or spironolactone (100 mg/day). Of the 20 AMAB participants, 12 received cyproterone acetate, 7 received spironolactone, and 1 had previously undergone an orchiectomy.

### Ethics approval

The study was approved by the Ethical Review Board of Istanbul University-Cerrahpaşa, Cerrahpaşa Medical School (Date: 04.04.2019; Reference Number: 04.04.2019-53259).

### Sample size

Sample size calculation was performed using PASS 11 software based on expected body weight changes in AMAB participants. With an anticipated effect size of 0.75 (10), 90% power, and an alpha error of 0.05, a minimum sample of 19 participants was required for the paired t-test analysis.

### Statistical analysis

Statistical analyses were performed using the Statistical Package for the Social Sciences (SPSS) software (version 21.0). Data were tested for normality using the Kolmogorov-Smirnov test. Continuous variables were expressed as mean ± standard deviation (SD) for normally distributed data and as median (interquartile range [IQR]) for non-normally distributed data. The Student’s t-test was used to compare groups with a normal data distribution, while medians were compared using the Mann-Whitney U and Kruskal-Wallis tests. For comparisons between Group 2 and Group 3, paired t-tests (for normally distributed data) or Wilcoxon signed-rank tests (for non-normally distributed data) were used to account for the within-subject design. Specific comparisons included cisgender women versus GAHT-naïve AMAB participants at treatment initiation (Group 1 *vs*. Group 2), cisgender women versus AMAB participants after 12 months of GAHT (Group 1 *vs*. Group 3), and within-subject changes in AMAB participants (Group 2 *vs*. Group 3). Correlations between variables were calculated using Spearman’s or Pearson’s correlation tests, depending on the data distribution. All results were analyzed using a 95% confidence interval, and statistical significance was defined as P < 0.05.

## Results

### Anthropometric and clinical characteristics

A total of 44 participants were included in the analysis: 24 cisgender women (Group 1) and 20 AMAB participants assessed at GAHT initiation (Group 2) and reassessed after 12 months of GAHT (Group 3). Physical examination findings and anthropometric measurements are summarized in **Table 1**. The groups were similar in age and body mass index (BMI). The waist-to-hip ratio was higher in AMAB participants than in cisgender women both at GAHT initiation and after 12 months of GAHT. Although arm and calf circumferences were slightly higher in AMAB participants, the absolute differences (approximately 1–2 cm) were small and likely of limited clinical relevance. AMAB participants demonstrated higher grip strength than cisgender women at GAHT initiation. After 12 months of GAHT, grip strength showed a non-significant decline.

**Table 1:** Demographic and anthropometric characteristics of cisgender women (Group 1), GAHT-naïve AMAB participants (Group 2), and the same AMAB participants after 12 months of GAHT (Group 3).

Variable	Cisgender women (Group 1, n=24)	GAHT-naïve AMAB participants (Group 2, n=20)	AMAB participants after 12 months of GAHT. (Group 3, n=20)	p-value 1 *vs* 2	p-value 1 *vs* 3	p-value 2 *vs* 3
Age (years) Mean ± SD [95% CI]	26.6 ± 4.5 [24.7–28.5]	26.0 ± 7.0 [22.7–29.2]	27.5 ± 7.5 [23.9–31.0]	0.20	0.70	0.40
BMI (kg/m²) Mean ± SD [95% CI]	21.5 ± 2.5 [20.5–22.6]	22.6 ± 3.8 [20.8–24.4]	22.8 ± 3.5 [21.5–24.4]	0.20	0.20	0.80
Systolic BP (mmHg) Mean ± SD [95% CI]	103 ± 25 [92-114]	117 ± 28 [104-130]	105 ± 34 [89-121]	0.90	0.90	0.90
Diastolic BP (mmHg) Mean ± SD [95% CI]	64 ± 12 [59-69]	68 ± 14 [61-75]	65 ± 10 [60-70]	0.80	0.90	0.80
Waist-to-Hip Ratio Mean ± SD [95% CI]	0.74 ± 0.06 [0.72–0.77]	0.85 ± 0.08 [0.82–0.89]	0.84 ± 0.08 [0.80–0.88]	<0.001	<0.001	0.40
Arm circumference (cm) Mean ± SD [95% CI]	25.6 ± 3.6 [24.2–27.3]	26.8 ± 3.9 [24.9–28.7]	26.3 ± 2.7 [25.0–27.7]	0.38	0.50	0.70
Calf circumference (cm) Median [IQR]	34 [32–36]	35.3 [33–40]	34.5 [32–37]	0.020	0.60	0.60
Gait speed (m/s) Median [IQR]	1.37 [1.33–1.45]	1.57 [1.30–1.80]	1.52 [1.30–1.80]	0.32	0.040	0.60
Muscle strength (kg) Mean ± SD [95% CI]	26.0 ± 3.4 [24.6–27.5]	35.6 ± 8.4 [31.3–39.8]	32.5 ± 6.6 [29.4–35.5]	<0.001	0.001	0.20

Values are presented as mean ± standard deviation (SD) with 95% confidence intervals (CI) for normally distributed variables, and as median with interquartile range (IQR) for non-normally distributed variables. Group comparisons were performed using Student’s *t*-test or Mann–Whitney *U* test (Group 1 *vs*. Group 2 and Group 1 *vs*. Group 3), and paired *t*-test or Wilcoxon signed-rank test (Group 2 *vs*. Group 3), according to data distribution.

AMAB, assigned male at birth; GAHT, gender-affirming hormone therapy.

### Bioimpedance analysis

**Figure 1:**
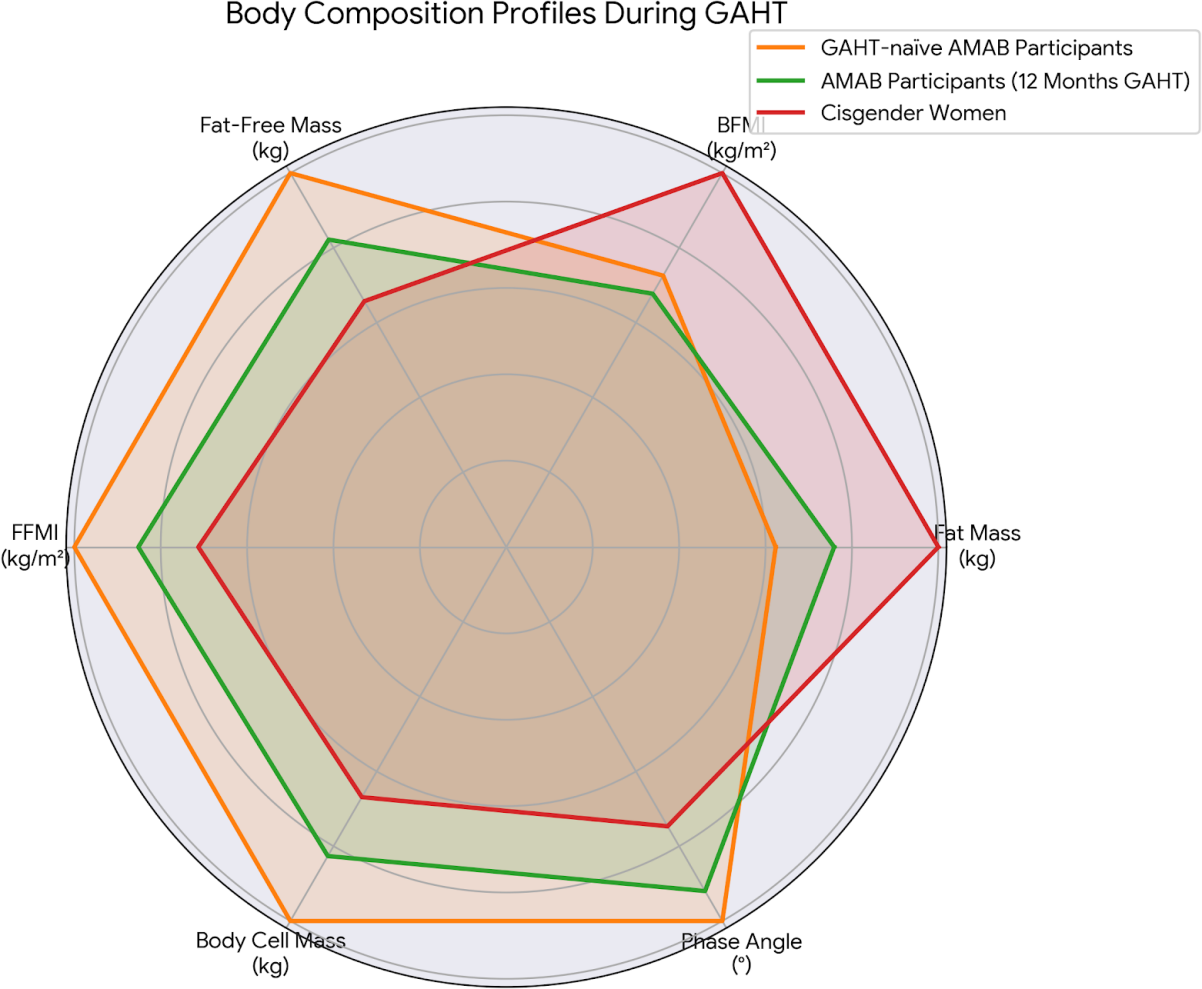
Radar chart illustrating the multidimensional shift in body composition profiles during gender-affirming hormone therapy (GAHT). Data values were normalized to the maximum observed value in each category to allow simultaneous comparison of mass (kg), indices (kg/m²), and phase angle (°). The chart displays the longitudinal changes of GAHT-naïve AMAB participants (Orange) to the 12-month follow-up (Green), compared against the cisgender women reference group (Red). The plot highlights the simultaneous reduction in lean mass parameters (Fat-Free Mass, Body Cell Mass, Phase Angle) toward the reference profile, while differences in fat mass and lean mass persist.

When comparing cisgender women (Group 1) with AMAB participants after 12 months of GAHT (Group 3), distinct differences were observed across several body composition parameters (**Table 2**, **Figure 1**). Fat mass remained higher in cisgender women (17.0 [13.8–21.3] kg) than in AMAB participants at the 12-month assessment (12.9 [10.8–16.1] kg; p = 0.009), and the body fat mass index (BFMI) showed a similar pattern (6.2 [5.3–7.1] *vs*. 4.2 [3.1–5.9] kg/m²; p < 0.001). Conversely, indices of lean mass remained significantly higher in AMAB participants after 12 months of GAHT: fat-free mass (FFM) (49.9 [39.4–54.6] *vs*. 39.9 [39.1–44.4] kg; p < 0.001), fat-free mass index (FFMI) (18.4 [16.5–19.4] *vs*. 15.4 [14.2–16.0] kg/m²; p < 0.001), body cell mass (BCM) (25.7 [20.6–29.3] *vs*. 20.8 [19.9–23.4] kg; p < 0.001), and phase angle (6.9° [6.0–7.6] *vs*. 5.6° [4.9–6.1]; p < 0.001). These results indicate that, despite reductions over the course of GAHT, AMAB participants retained greater lean mass, cellular mass, and membrane integrity compared with cisgender women.

**Table 2:** Bioelectrical impedance analysis (BIA) parameters of cisgender women, GAHT-naïve AMAB participants, and AMAB participants after 12 months of GAHT.

Variable	Cisgender women (Group 1, n=24)	GAHT-naïve AMAB participants (Group 2, n=20)	AMAB participants after 12 months of GAHT (Group 3, n=20)	p-value 1 *vs* 2	p-value 1 *vs* 3	p-value2 *vs* 3	% Change (G2 → G3) [95% CI]
Fat mass (kg) Median [IQR]	17 [13.8-21.3]	10.6 [8.9-13.8]	12.9 [10.8-16.1]	<0.001	0.009	0.07	+28.7% [+4.7% to +52.7%]
BFMI (kg/m²) Median [IQR]	6.2 [5.3-7.1]	4.5 [3.4-5.1]	4.2 [3.1-5.9]	<0.001	<0.001	0.08	–18.6% [–9.8% to +47.0%]
Fat-free mass (kg) Median [IQR]	39.9 [39.1-44.4]	60.7 [47.6-68.3]	49.9 [39.4-54.6]	<0.001	<0.001	0.01	–17.1% [–26.7% to –7.4%]
FFMI (kg/m²) Median [IQR]	15.4 [14.2-16.0]	21.6 [18.0-24.0]	18.4 [16.5-19.4]	<0.001	<0.001	0.04	–10.6% [–19.7% to –1.4%]
Body cell mass (kg) Median [IQR]	20.8 [19.9-23.4]	31.1 [23.9- 34.1]	25.7 [20.6-29.3]	<0.001	<0.001	0.04	–10.5% [–22.4% to +1.3%]
Phase angle (°) Median [IQR]	5.6 [4.9-6.1]	7.5 [6.7-8.3]	6.9 [6.0-7.6]	<0.001	<0.001	0.04	–7.9% [–15.4- –0.4]

Values are presented as mean ± SD or median [IQR], as appropriate. Between-group comparisons were conducted using Student’s *t*-test or Mann–Whitney *U* test, and within-subject changes (Group 2 *vs*. Group 3) were analyzed using paired *t*-tests or Wilcoxon signed-rank tests. BFMI, body fat mass index; FFMI, fat-free mass index; AMAB, assigned male at birth; GAHT, gender-affirming hormone therapy.

Comparisons between cisgender women and GAHT-naïve AMAB participants (Group 2) revealed similar contrasts. Cisgender women had higher fat mass (17.0 *vs*. 10.6 kg; p < 0.001; d = 1.70) and BFMI (6.2 *vs*. 4.5 kg/m²; p < 0.001; d = 1.61), whereas GAHT-naïve AMAB participants showed higher FFM (60.7 *vs*. 39.9 kg; p < 0.001; d = –1.99), FFMI (21.6 *vs*. 15.4 kg/m²; p < 0.001; d = –2.48), BCM (31.1 *vs*. 20.8 kg; p < 0.001; d = –1.82), and phase angle (7.5° *vs*. 5.6°; p < 0.001; d = –2.51).

In the longitudinal analysis (Group 2 → Group 3), GAHT was associated with measurable within-subject changes over one year. Fat mass showed an increasing trend (10.6 [8.9–13.8] → 12.9 [10.8–16.1] kg; +28.7%; p = 0.07; d = –0.74), while BFMI showed minimal change (4.5 [3.4–5.1] → 4.2 [3.1–5.9] kg/m²; p = 0.08; d = –0.22). Lean-related parameters declined significantly during the same period: FFM decreased from 60.7 to 49.9 kg (p = 0.01; d = 1.00), FFMI from 21.6 to 18.4 kg/m² (p = 0.04; d = 0.89), BCM from 31.1 to 25.7 kg (p = 0.04; d = 0.82), and phase angle from 7.5° to 6.9° (p = 0.04; d = 0.80).

Correlation analyses supported these findings. Phase angle was positively associated with BMI (r = 0.7, p < 0.001), FFM (r = 0.5, p < 0.001), FFMI (r = 0.6, p < 0.001), and BCM (r = 0.6, p < 0.001).

### Muscle strength

At GAHT initiation, AMAB participants exhibited significantly higher muscle strength compared to cisgender women (p < 0.001, **Table 1**). Although muscle strength tended to decline after 12 months of GAHT, this reduction did not reach statistical significance (p = 0.20), and the within-subject effect size suggested only a modest change over time (d = 0.41). Importantly, even after 12 months of treatment, muscle strength in AMAB participants remained significantly higher than in cisgender women (p = 0.001), with the between-group difference still reflecting a substantial effect (d = 1.27).

### Gait speed

At GAHT initiation, no statistically significant difference in walking speed was observed between AMAB participants and cisgender women (1.57 m/s *vs*. 1.37 m/s; p = 0.32). Over 12 months of GAHT, gait speed in AMAB participants remained stable (1.52 m/s; p = 0.60 *vs*. GAHT initiation). However, at the 12-month assessment, a significant between-group difference was observed, with AMAB participants walking faster than cisgender women (1.52 m/s *vs*. 1.37 m/s; p = 0.04).

### Laboratory results

After 12 months of GAHT, estradiol concentrations increased into the female reference range in all AMAB participants, while testosterone levels were effectively suppressed to <50 ng/dL. Hormone levels at follow-up were comparable to those observed in cisgender women. Other metabolic parameters, including hemoglobin A1c (HbA1c) and lipid profiles, remained stable throughout the follow-up period (**Table 3**).

**Table 3:** Hormonal and biochemical parameters at GAHT initiation and after 12 months of GAHT.

Laboratory parameter	Cisgender women (Group 1, n=24)	GAHT-naïve AMAB participants (Group 2, n=20)	AMAB participants after 12 months of GAHT. (Group 3, n=20)	p-value1 *vs* 3
Estradiol (pg/mL) Mean ± SD [95% CI]	104 ± 17 [98-110]	31± 8 [26-37]	90 ± 12 [82–98]	0.1
Testosterone (ng/dL) Mean ± SD [95% CI]	30 ± 7 [26-37]	561± 292 [518-602]	40 ± 6 [32-46]	0.3
LH (mIU/mL) Median [IQR]	5.2 ± 0.3 [4.8–5.4]	5.2± 2.6 [4.8–5.6]	4 ± 0.8 [3.9–4.3]	0.1
FSH (mIU/mL) Mean ± SD [95% CI]	3.1 ± 0.2 [2.8–3.5]	5 ± 5 [4.7–5,2]	2.4 ± 0.3 [2.0–2.8]	0.2
Prolactin (µg/L) Mean ± SD [95% CI]	16 ± 4 [13-19]	25± 6 [17-30]	24 ± 3 [20-27]	0.3
HbA1c (%) Mean ± SD [95% CI]	5.4 ± 0.7 [5.2-5.7]	5.2± 0.2 [4.8-5.5]	5.5 ± 0.5 [5.1-5.9]	0.9
Total Cholesterol (mg/dL) Mean ± SD [95% CI]	200 ± 55 [176.8 – 223.2]	183 ± 49 [160.1 – 205.9]	171 ± 33 [155.6 – 186.4]	0.5
LDL (mg/dL) Mean ± SD [95% CI]	102 ± 32 [93-111]	100± 38 [90-115]	109 ± 40 [100-118]	0.8
HDL (mg/dL) Mean ± SD [95% CI]	56 ± 12 [52-60]	54± 14 [51-59]	54 ± 12 [50-57]	0.6
Triglycerides (mg/dL) Mean ± SD [95% CI]	73 [57-113]	55[47-115]	69 [55-214]	0.8

Values are presented as mean ± standard deviation (SD) or median with interquartile range (IQR), depending on data distribution. Hormonal comparisons in this table were performed only between cisgender women (Group 1) and AMAB participants after 12 months of GAHT (Group 3). Hormone levels measured in GAHT-naïve AMAB participants (Group 2) represent pre-treatment values at GAHT initiation and were not included in between-group statistical comparisons. LH, Luteinizing Hormone; FSH, Follicle-Stimulating Hormone; GAHT, Gender-affirming hormone therapy.

## Discussion

Our findings align with established literature regarding the effects of gender-affirming hormone therapy (GAHT) on body composition and muscle-related parameters in individuals with gender dysphoria. Previous meta-analyses, such as those by Klaver et al. (10) and Gois et al. (14), have consistently reported that feminizing GAHT is associated with increases in fat mass and reductions in lean body mass. Similarly, Harper et al. (8) documented reductions in muscle strength, lean body mass, and muscle cross-sectional area in AMAB participants receiving GAHT, although these parameters remained higher than those observed in cisgender women.

In contrast, comparative studies highlight divergent responses based on sex assigned at birth. Wiik et al. (11) reported significant gains in muscle mass and strength in individuals assigned female at birth (AFAB) after one year of testosterone therapy, whereas only marginal changes were observed in AMAB participants receiving feminizing therapy. A recent meta-analysis further underscored this divergence, demonstrating significant increases in lean mass (+4.12 kg) and decreases in fat mass (–1.29 kg) in AFAB cohorts (15). In our study, AMAB participants showed significant decreases in lean body mass, consistent with these expected physiological adaptations rather than pathological alterations (12, 14).

Bioimpedance-derived parameters, specifically phase angle (PhA) and body cell mass (BCM), remain underutilized in the evaluation of body composition during GAHT. In the present study, both PhA and BCM values were significantly lower after 12 months of GAHT compared with GAHT initiation values, indicating therapy-related changes in body composition that may have longer-term clinical relevance (16). The observed decrease in PhA likely reflects the physiological redistribution of body compartments toward a female-typical pattern rather than cellular deterioration. Notably, despite this decline, PhA values remained within the healthy range and continued to exceed those observed in cisgender women. This supports the interpretation of these changes as part of a female-typical physiological adaptation rather than evidence of functional impairment (17, 18). Finally, the absence of a correlation between changes in PhA and grip strength suggests that these parameters reflect parallel but independent physiological processes, underscoring the importance of interpreting each measure within its specific biological context.

Regarding adiposity, estrogen-based GAHT has historically been linked to increases in total body fat mass in AMAB participants (10, 19, 20). In the present study, although fat mass and body fat mass index (BFMI) showed an increasing trend after 12 months, these changes did not reach statistical significance. Nevertheless, cisgender women continued to exhibit higher fat mass and BFMI values than AMAB participants even after 12 months of therapy. Given that participants achieved target hormone levels—with estradiol concentrations comparable to cisgender women and testosterone effectively suppressed—the modest changes in fat mass observed here likely reflect the gradual, time-dependent nature of adipose tissue redistribution, which may require longer durations of GAHT to fully manifest.

In terms of physical performance, previous research by Roberts et al. noted that while the difference in running speed between AMAB participants and cisgender women narrowed following hormone therapy, AMAB individuals remained approximately 9% faster even after prolonged testosterone suppression (21). Our findings support this observation. At GAHT initiation, no significant difference in gait speed was observed between groups (p = 0.32). Over 12 months, gait speed in AMAB participants remained stable (1.52 m/s), yet a significant between-group difference emerged at the 12-month assessment, with AMAB participants walking faster than cisgender women (p = 0.04). Since height and leg length are critical determinants of walking speed and remain unaffected by hormonal treatment (22), the persistence of faster gait speeds in our cohort suggests that biomechanical factors may contribute to preserved functional performance even as muscle physiology undergoes hormonally mediated adaptation.

Metabolic health remains a critical consideration, as increased fat mass in AMAB participants has been associated with insulin resistance (23). In this study, no overt metabolic complications were observed, which may be attributed to regular clinical monitoring, dietary guidance, and the relatively short duration of follow-up. However, a modest, non-significant increase in HbA1c levels (from 5.2% to 5.5%) was noted, underscoring the importance of continued metabolic monitoring during ongoing GAHT. Additionally, although statistically significant between-group differences were observed for certain anthropometric parameters, small absolute variations in measurements such as arm or calf circumference (approximately 1–2 cm) fall within the expected range of normal biological variability and are likely of limited clinical relevance.

### Limitations

This study has several limitations that should be considered when interpreting the findings. First, the sample size was modest, which limits statistical power and the generalizability of the results. Second, cisgender women were assessed at a single time point rather than followed longitudinally; consequently, they served as a reference for female-typical values rather than a true longitudinal control. This design limits the ability to attribute observed changes exclusively to GAHT, as factors such as aging or lifestyle changes could not be controlled for in the reference group. Third, although participants received routine clinical monitoring and nutritional guidance, day-to-day variability in dietary intake and physical activity could not be fully quantified. Fourth, bioelectrical impedance analysis (BIA) provided a practical non-invasive assessment but possesses lower precision than dual-energy X-ray absorptiometry (DXA), restricting direct comparability with imaging-based studies. Finally, heterogeneity in GAHT regimens specifically the use of cyproterone acetate versus spironolactone, and one case of orchiectomy may have introduced variability, particularly regarding fluid balance and PhA. Due to the small cohort size, subgroup analyses to further explore these regimen-specific differences were not feasible. For these reasons, the findings should be considered exploratory.

## Conclusion

This comparative longitudinal study demonstrates that AMAB participants undergoing GAHT exhibit measurable changes in body composition over 12 months, including reductions in lean body mass, fat-free mass index (FFMI), body cell mass (BCM), and phase angle (PhA). These changes are consistent with expected physiological adaptation toward female-typical body composition patterns rather than pathological muscle loss. Notably, although several parameters shifted toward values observed in cisgender women, they did not fully converge within the 12-month period, indicating that the feminization of body composition is a gradual process that likely extends beyond the initial year of treatment.

The observed reduction in PhA—while remaining higher than values in cisgender women—highlights the utility of bioelectrical impedance analysis and supports the role of BCM and PhA as valuable yet underutilized markers for monitoring compositional changes alongside conventional anthropometric measures. Establishing baseline measurements and implementing comprehensive longitudinal monitoring are therefore essential to ensure that these adaptations remain within healthy physiological ranges. Future studies with larger cohorts and longer follow-up durations are needed to clarify the long-term clinical and metabolic significance of these changes.

## Data Availability

The raw data supporting the conclusions of this article will be made available by the authors, without undue reservation.
